# Prognostic and Clinicopathological Significance of Hypoxia-Inducible Factor-1α in Endometrial Cancer: A Meta-Analysis

**DOI:** 10.3389/fonc.2020.587420

**Published:** 2020-11-11

**Authors:** Ping Zhu, Longxia Shen, Qiuxia Ren, Qingxiang Zeng, Xiaocui He

**Affiliations:** Department of Gynaecology and Obstetrics, Heze Municipal Hospital, Heze City, China

**Keywords:** endometrial cancer, prognosis, HIF-1α, immunohistochemistry, meta-analysis

## Abstract

**Background:**

Current reports on the prognostic and predictive value of hypoxia-inducible factor-1α (HIF-1α) in endometrial carcinoma are inconsistent. Therefore, we conducted this meta-analysis to precisely evaluate the association of HIF-1α expression with susceptibility, clinical features, and prognosis of endometrial cancer.

**Methods:**

Eligible studies that assessed the role of HIF-1α protein expression, immunohistochemistry detection, disease susceptibility, clinical features, and prognosis of endometrial cancer were searched from the Embase, Pubmed, and Web of Science databases. Stata 14.0 software was used to merge and compute pooled hazard ratios (HR) and odds ratios (OR). Information including HIF-1α protein expression and clinical progression of endometrial cancer was extracted. The pooled HR and OR with corresponding 95% confidence intervals (CI) were used to estimate the strength of these associations.

**Results:**

A total of 25 studies were included in the analysis. HIF-1α protein expression in endometrial cancer tissue was significantly higher than that in normal tissues (OR = 15.79, 95% CI = 8.44–29.52, *P* < 0.05). Endometrial cancer patients with higher HIF-1α protein expression had poorer prognosis compared to patients with low HIF-1α protein expression (HR = 2.29, 95% CI = 1.68–2.90, *P* < 0.05). In addition, high HIF-1α protein expression was significantly associated with endometrial cancer grade, lymph node metastasis, and myometrial invasion (grade in Caucasians: OR = 3.09, 95% CI = 1.63–5.85, *P* < 0.05; lymph node metastasis: OR = 3.09, 95% CI = 1.63–5.85, *P* < 0.05; myometrial invasion: OR = 2.26, 95% CI = 2.15–5.08, *P* < 0.05).

**Conclusions:**

HIF-1α overexpression was significantly associated with increased risk, advanced clinical progression, and poor prognosis in endometrial cancer patients.

## Introduction

According to Global Cancer Statistics 2018, endometrial cancer is the sixth most common tumor and the 11th leading cause of death in women worldwide with 382,069 new cases and 89,929 deaths in 2018 (1). Endometrial cancer is clinically divided into two categories: type I (95%) and type II (15%) (2, 3). Type I endometrial cancer is a low-grade endometrioid tumor that is commonly confined to the uterus, and patient survival rates after surgery are high. However, type II cancer which includes papillary serous tumors, clear cell tumors, and carcinosarcomas is more invasive and have a poor prognosis than type I cancer (4). In addition to surgery, targeted therapy for endometrial cancer has been developed in recent years. The angiogenesis pathway, PI3K/Akt/mTOR pathway, and glucose metabolism have been targeted by drugs, such as bevacizumab, ridaforolimus, and metformin for endometrial cancer treatment (5–7). However, there is scope for the development of newer biomarkers for detection as well as drugs that target other biological pathways. In 2013, the Cancer Genome Atlas Research Network published a study in the *Nature* journal, reporting on the integrated genomic, transcriptomic, and proteomic characterization of 373 endometrial carcinomas using array- and sequencing-based technologies. The authors classified endometrial cancers into four categories: copy-number high (with poor 5-year progression-free survival rate), DNA-polymerase epsilon (POLE) (ultra-mutated, with >95% progression-free survival rate), microsatellite instability hypermutated, and copy-number low (microsatellite stable) (8). This reclassification reveals that different endometrial carcinomas had different molecular characteristics that might affect postsurgical adjuvant treatment for women with aggressive tumors (8). In this classification, each group of patients had a different BMI, which might suggest that obesity affects the genetic characteristics of endometrial tumors (9). In addition to BMI, hyperglycemia, hypoxia, and glucose metabolism had significant associations with endometrial cancer. Glucose metabolism in endometrial tumor cells significantly increased compared to normal cells and facilitated the proliferation and growth of tumor cells (10). Therefore, although many early-stage cancer patients are responsive to radiotherapy and chemotherapy, some tumors often recur because of tumor cell proliferation and angiogenesis (11). The rapid proliferation and growth of tumor cells and the development of tumor blood vessels often lead to local hypoxia. However, tumor cells are able to acquire sufficient energy to sustain physiological activity in the hypoxic environment, and this mechanism is closely associated with the activation of hypoxia-related genes, such as hypoxia-inducible factor-1α (HIF-1α).

HIF-1α is a transcription factor that plays a crucial role in the adaptive cellular response to hypoxia. HIF-1α regulates several biological processes, such as glucose metabolism, gluconeogenesis, high-energy phosphate metabolism, cell growth, apoptosis, erythropoiesis, heme metabolism, iron transport, vasomotor regulation, and nitric oxide synthesis (12). For example, the activity of glucose transporters (GLUTs), which are responsible for glucose uptake, is regulated by HIF-1α (13). The upregulation of GLUT1 induces the shift in glucose metabolism toward glycolysis in hypoxia. In addition, HIF-1α regulates the pH of the tumor microenvironment by promoting the expression of carbon anhydrase IX (CAIX) (14). In recent years, researchers have conducted many studies to investigate the role of HIF-1α in endometrial cancer to evaluate the predictive and prognostic value of HIF-1α protein expression. We retrieved relevant studies from databases and found that they were few in number, and their results were conflicting. Thus, we performed this meta-analysis to assess the association of HIF-1α protein expression with endometrial cancer.

## Materials and Methods

### Literature Search Strategy

We searched for relevant articles published up to May 2, 2020, using the Pubmed, Embase, and Web of Science databases. The search terms used were “endometrial cancer,” “HIF-1α,” “carcinoma of endometrium,” “endometrial neoplasms,” “hypoxia-inducible factor,” and “prognosis.” In addition, the references of relevant literatures were scanned to collect other eligible articles.

### Inclusion and Exclusion Criteria

To collect all eligible articles and improve retrieval efficiency, the following standard inclusion criteria were developed: (1) articles that included the association between endometrial cancer and HIF-1α protein expression; (2) patients included were diagnosed by pathological examination; (3) the expression of HIF-1α protein was detected by immunohistochemistry (IHC); and (4) relevant data, such as hazard ratios (HR) and 95% confidence intervals (CI), survival curve, and clinical information, could be obtained from the article. Articles were excluded if they were (1) reviews, letters, case reports, meta-analyses, editorials, conference abstracts, or animal trials or (2) repeat studies based on the same data or cancer patients.

### Data Extraction and Quality Assessment

Two investigators independently extracted data from eligible studies, including authors’ names, publication date, race, country, method of protein detection, HR and 95% CI of survival rate, survival curves, and cutoff value of protein detection. We applied the Newcastle-Ottawa Scale (NOS) to assess the quality of included studies and those with a score ≥ 6 were considered as high-quality studies (15). Any discrepancy in the data extraction process was resolved by consensus between investigators.

### Statistical Analysis

All statistical analyses were conducted using the Stata 14.0 and Engauge Digitizer 4.1 software. The pooled HR and 95% CI were used to evaluate the role of HIF-1α protein overexpression in the prognosis of endometrial cancer patients. Further, the pooled odds ratios (OR) and 95% CI were used to assess the association of HIF-1α protein overexpression with risk and clinical features of endometrial cancer (16). If the article did not provide the HR and 95% CI, it was extracted from the survival curve using Engauge Digitizer 4.1. Chi-square test and *I^2^* statistic were used to calculate the pooled OR and 95% CI (17). Values of *I^2^* ≥ 50% or *P* <0.05 revealed that significant heterogeneity existed among studies, and the random-effect model was used. Otherwise, the fixed-effect model was applied (18). The Begg test and Egger test were used to examine any underlying publication bias (19). In all tests, *P* <0.05 was considered as significant.

## Results

### Study Characteristics

**Figure 1:**
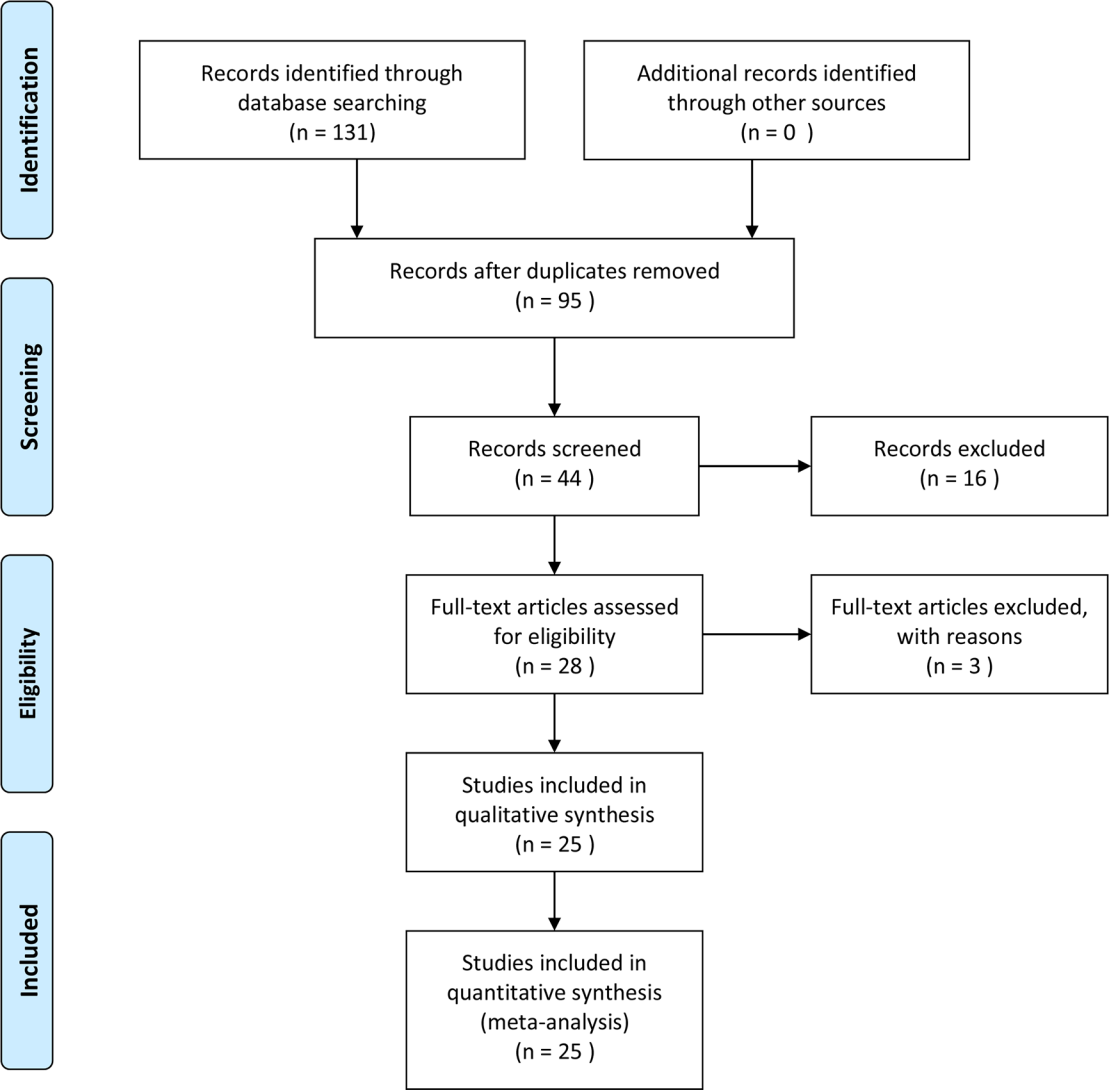
Flowchart of the search protocol for eligible studies.

We searched the Pubmed, Embase, and Web of Science databases separately, and 131 articles were retrieved in the initial search. Duplicates were removed, and 95 articles were obtained. After we read the titles and abstracts of these articles, 16 studies were deemed not related to endometrial cancer or HIF-1α protein expression and were removed. In the remaining 28 studies, the full text was carefully screened, and 3 studies were eliminated because of no available data. Based on the inclusion criteria, 25 studies were included finally (20–44), among which 6 studies were on the survival of endometrial cancer patients and all 25 studies included risk and clinical features of endometrial cancer patients. The search protocol is depicted in **Figure 1**, and the information extracted from the articles is given in **Tables 1** and **2**. The quality of included studies is also provided in **Table 1**. The quality score for the included studies on the relationship between HIF-1α expression and endometrial cancer was ≥6, and they were considered as high quality.

**Table 1:** Eligible studies for the risk of endometrial cancer and HIF-1α expression.

Author	Reference	Time	Country	Ethnicity	Method	Cancer type	Histology	Normal tissue		Cancer tissue		Cut-off value	NOS
								HIF-1α -	HIF-1α +	HIF-1α -	HIF-1α +		
Feng	(20)	2006	China	Asians	IHC	EEC	Tissue	15	0	21	29	0%	6
Wang	(21)	2007	China	Asians	IHC	EEC	Tissue	11	3	16	28	10%	6
Horree	(22)	2007	Netherlands	Caucasians	IHC	EEC	Tissue	17	0	5	34	5%	7
Zhang	(23)	2010	China	Asians	IHC	EEC	Tissue	16	1	9	30	5%	6
Feng	(24)	2013	China	Asians	IHC	EEC	Tissue	26	9	43	81	0%	6
Chen	(25)	2015	China	Asians	IHC	EEC	Tissue	19	4	23	35	0%	6
Wu	(26)	2016	China	Asians	IHC	EEC	Tissue	37	3	24	41	5%	6
Zhu	(27)	2019	China	Asians	IHC	EEC	Tissue	25	5	9	41	0%	6
Sun	(28)	2019	China	Asians	IHC	EEC	Tissue	114	14	32	96	NR	6

IHC, immunohistochemistry; EEC, endometrioid endometrial carcinoma.

**Table 2:** Included studies for the survival of endometrial cancer and HIF-1α expression.

Author	Reference	Time	Country	Ethnicity	Tumor stage	Detected sample	Num.	Follow-up median	Method	Survival analysis	Source of HR	HR	LL	UL	P	Cut-off	95%CI
Abouhashem	(29)	2016	Egypt	Mixed	IHC	ECT	50	12		OS	Curve	3.36	0.84	6.02	0.097	10%	0.84-6.02
Berg	(30)	2016	Norway	Caucasians	IHC	ECT	86	30		OS	HR	2.17	1.37	3.49	0.004	NR	1.37-3.49
Seeber	(31)	2010	Netherlands	Caucasians	IHC	ECT	54	95		OS	Curve	2.53	0.78	4.28	0.235	NR	0.78-4.28
Sivridis	(32)	2002	UK	Caucasians	IHC	ECT	81	75		OS	Curve	1.86	1.04	3.64	0.03	NR	1.04-3.64
Aybatli	(33)	2012	Turkey	Caucasians	IHC	ECT	76	56		OS	Curve	1.85	0.83	3.61	0.222	0%	0.83-3.61
Soo	(34)	2017	Korea	Asians	IHC	ECT	140	104		OS	HR	3.79	2.18	6.35	0.001	0%	2.18-6.35

ECT, endometrial carcinoma tissue; IHC, immunohistochemistry; OS, overall survival; HR, hazard ratio; NR, not reported.

### Meta-Analysis of Patient Survival 

**Figure 2:**
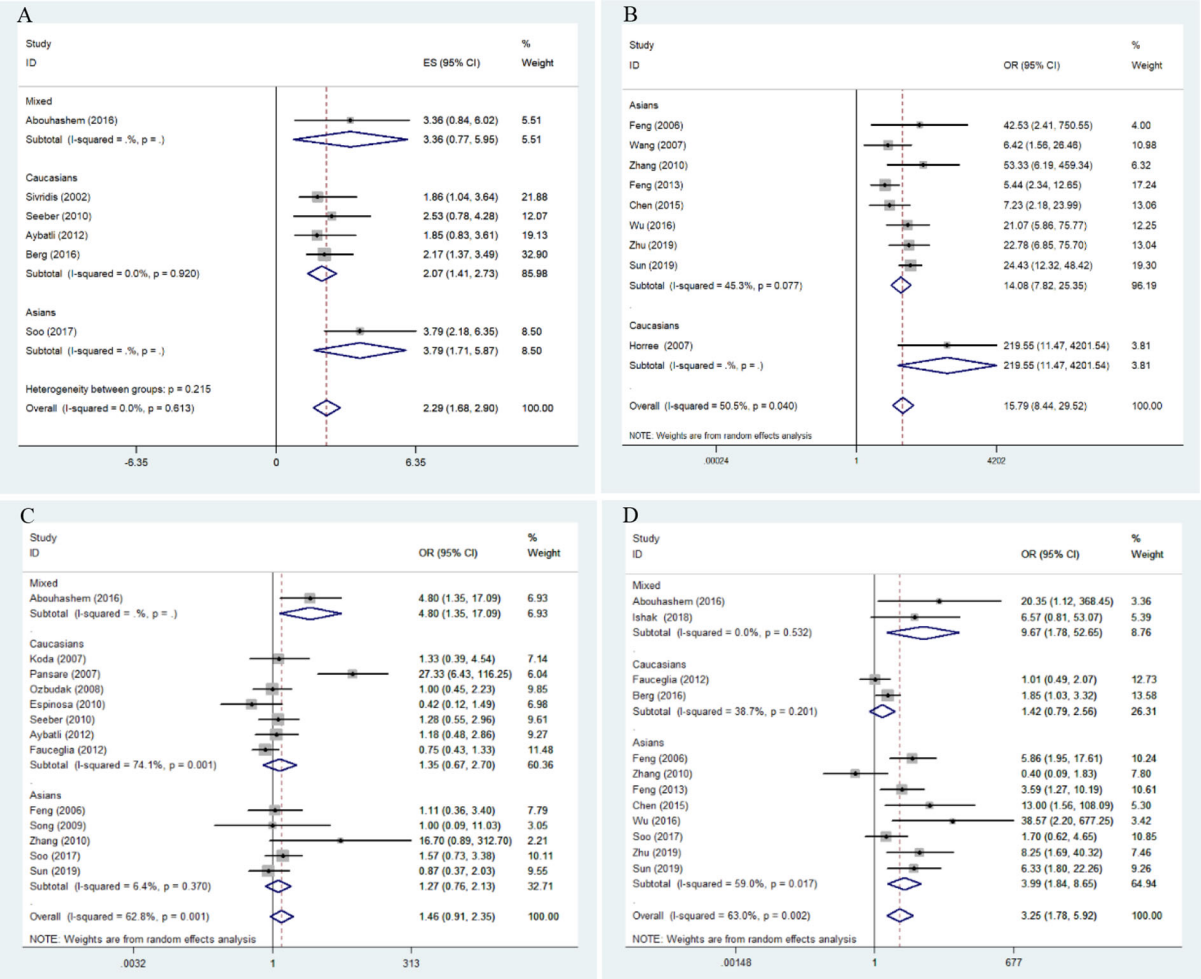
Forest plots for the association of risk, clinical features, and overall survival in endometrial cancer with HIF-1α expression. OR, odds ratio; CI, confidence interval; **(A)** overall survival in endometrial cancer; **(B)** risk of endometrial cancer; **(C)** stage of endometrial cancer; **(D)** lymphatic metastasis of endometrial cancer.

**Figure 3:**
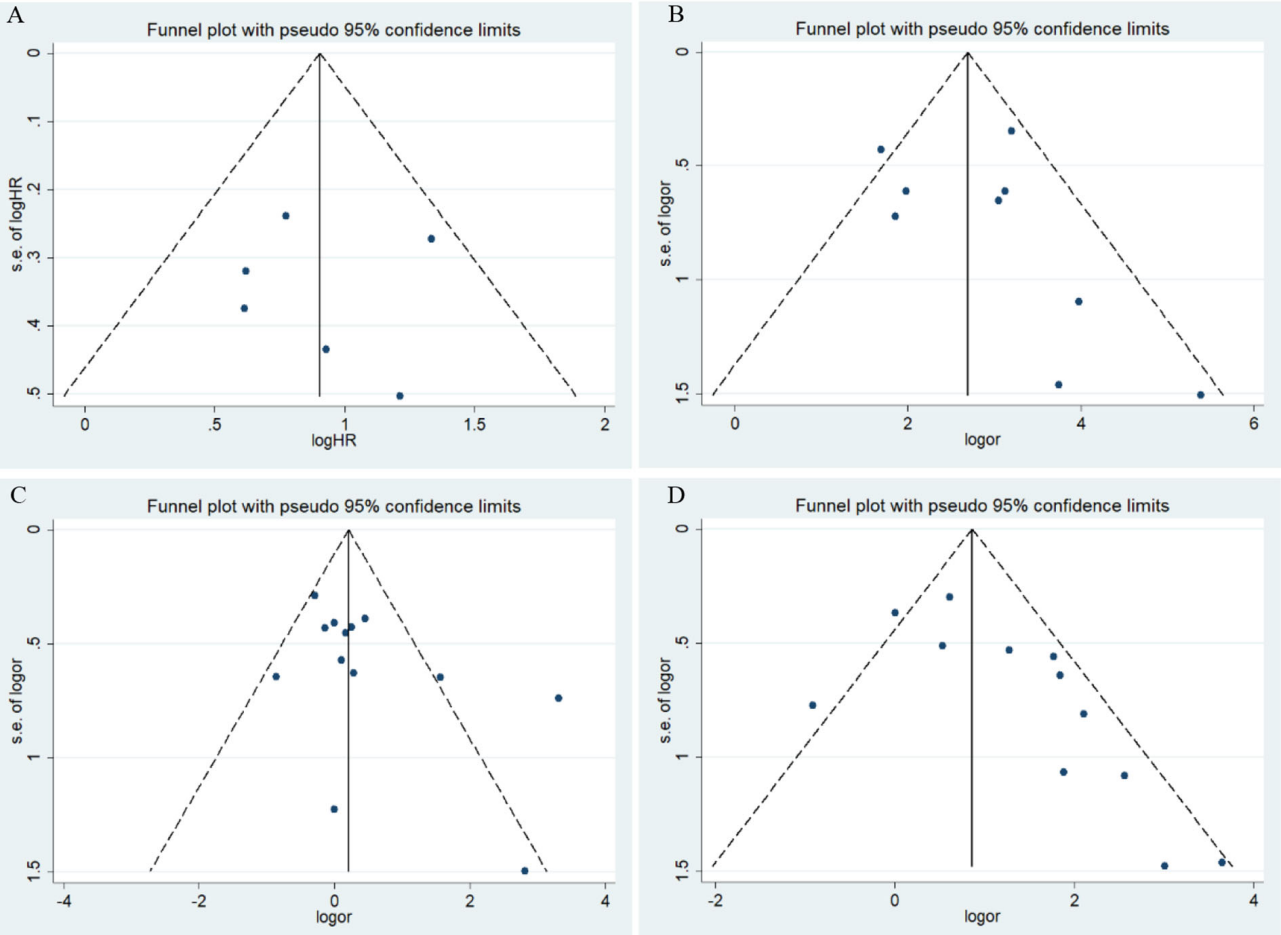
Funnel plots for the association of risk, clinical features, and overall survival in endometrial cancer with HIF-1α expression. OR, odds ratio; CI, confidence interval; **(A)** overall survival in endometrial cancer; **(B)** risk of endometrial cancer; **(C)** stage of endometrial cancer; **(D)** lymphatic metastasis of endometrial cancer.

Overall results indicate that HIF-1α protein overexpression predicts a poor prognosis in endometrial cancer patients (HR = 2.29, 95% CI = 1.68–2.90, *P* <0.05). This result is applicable to both Caucasians (HR = 2.07, 95% CI = 1.41–2.73, *P* <0.05) and Asians (HR = 3.79, 95% CI = 2.18–6.35, *P* <0.05). No significant heterogeneity was found among studies, and the results of the Begg and Egger tests did not indicate any obvious publication bias. However, only one study was included in the meta-analysis of overall survival of endometrial cancer patients (**Figures 2A** and **3A**).

### Meta-Analysis of Clinicopathological Parameters

Meta-analysis results for the association of HIF-1α protein overexpression and risk as well as clinicopathological parameters are presented in **Table 3**. Significant heterogeneity is found in some analyses for clinical features of endometrial cancer patients (**Table 3**). Here, the random-effect model was used to calculate the pooled OR and 95% CI. First, HIF-1α protein overexpression was significantly associated with the risk of endometrial cancer (OR =15.79, 95% CI = 8.44–29.52, *P* <0.05) (**Figure 2B**). In the subgroup analysis based on race as a risk factor for endometrial cancer, there was no heterogeneity. We also find a significant association between race and risk for cancer in Asians (OR =14.08, 95% CI = 7.82–25.35, *P* <0.05). Moreover, the results reveal that high HIF-1α protein expression is associated with advanced tumor grade in Caucasians (Caucasians: OR =3.09, 95% CI = 1.63–5.85, *P* <0.05), and HIF-1α protein overexpression clearly promotes lymph node metastasis (Asians: OR =3.99, 95% CI = 1.84–8.65, *P* <0.05) (**Figure 2D**) and myometrial invasion (Asians: OR =3.30, 95% CI = 2.15–5.08, *P* <0.05) of endometrial tumor cells in Asians. Although several studies explore the association of HIF-1α with the TNM (T: primary tumor; N: regional lymph nodes; M: distant metastasis) stage (**Figure 2C**), PR status, ER status, recurrence, and type of endometrial cancer (endometrioid type and serous type), no significant difference is found in the pooled overall results. The results indicate that some publication bias exists among studies for some analyses of risk, lymph node metastasis, recurrence, and myometrial invasion of endometrial cancer (*P* <0.05). However, this publication bias markedly decreases in the subgroup analysis (**Figures 3B–D**). Sensitivity analysis suggests that no individual study significantly affects the overall results; therefore, there was no need to remove a study.

**Table 3:** Meta results for the association between HIF-1α expression and endometrial cancer.

Characteristics (Negative vs Positive)	Studies	Pooled OR (95% CI)	*P*	Heterogeneity		Begg’s test		Egger’s test	
				I^2^ (%)	*P*	Z	*P*	T	*P*
Risk (Overall)	9	15.79 (8.44, 29.52)	<0.05	50.50%	0.04	1.15	0.25	0.99	0.36
Risk (Asian)	8	14.08 (7.82, 25.35)	<0.05	45.30%	0.08	0.37	0.71	0.38	0.72
Tumor grade (Overall) (G1 vs G2, G3)	15	1.78 (0.97, 3.26)	>0.05	74.70%	0.00	0.99	0.32	-0.57	0.58
Tumor grade (Caucasian) (G1 vs G2, G3)	4	3.09 (1.63, 5.85)	<0.05	14.10%	0.32	0.31	0.75	-0.38	0.47
Tumor grade (Asian) (G1 vs G2, G3)	9	1.21 (0.50, 2.91)	<0.05	80.30%	0.00	1.70	0.09	-6.09	0.03
Lymph node metastasis (Overall) (N0 vs N1)	12	3.25 (1.78, 5.92)	<0.05	63.00%	0.00	1.99	0.05	2.64	0.03
Lymph node metastasis (Caucasian) (N0 vs N1)	2	1.42 (0.79, 2.56)	>0.05	38.70%	0.20	0.00	1.00	–	–
Lymph node metastasis (Asian) (N0 vs N1)	8	3.99 (1.84, 8.65)	<0.05	59.00%	0.02	2.10	0.04	1.08	0.32
TNM stage (Overall) (T1 vs T2-T4)	13	1.46 (0.91, 2.35)	>0.05	62.80%	0.00	1.53	0.13	2.01	0.07
TNM stage (Caucasian) (T1 vs T2-T4)	7	1.35 (0.67, 2.70))	>0.05	74.10%	0.00	1.20	0.23	1.45	0.21
TNM stage (Asian) (T1 vs T2-T4)	5	1.27 (0.76, 2.13)	>0.05	6.40%	6.40	0.73	0.46	1.02	0.38
FIGO ((Ⅰ+Ⅱ) vs (Ⅲ+Ⅳ))	8	2.88 (0.89, 8.03)	>0.05	88.90%	0.00	0.87	0.39	1.11	0.31
FIGO ((Ⅰ+Ⅱ) vs (Ⅲ+Ⅳ)) (Caucasians)	3	3.38 (0.50, 22.77)	>0.05	95.00%	0.00	1.04	0.30	2.55	0.24
FIGO ((Ⅰ+Ⅱ) vs (Ⅲ+Ⅳ)) (Asians)	4	2.38 (0.35, 16.02)	>0.05	85.50%	0.00	-0.34	1.00	-0.33	0.77
Myometrial invasion (<50% vs >50%)	11	2.26 (1.70, 3.01)	<0.05	39.70%	0.08	1.71	0.09	2.81	0.02
Myometrial invasion (Caucasian) (<50% vs >50%)	3	1.18 (0.75, 1.86)	>0.05	0.00%	0.93	1.04	0.30	7.14	0.09
Myometrial invasion (Asian) (<50% vs >50%)	6	3.30 (2.15, 5.08)	<0.05	0.00%	0.55	0.75	0.45	1.26	0.28
PR	3	1.59 (0.29, 8.69)	>0.05	91.10%	0.00	0.00	1.00	1.80	0.32
ER	3	0.92 (0.28, 3.03)	>0.05	79.40%	0.01	1.04	0.30	1.93	0.30
Recurrence	3	2.71 (0.74,9.99)	>0.05	73.00%	0.03	1.04	0.30	21.95	0.03
Type1 vs Type2	4	1.38 (0.19, 9.84)	>0.05	93.70%	0.00	-0.34	1.00	-0.03	0.98
		Pooled HR (95% CI)							
OS	6	2.29 (1.68, 2.90)	<0.05	0%	0.613	0.38	0.707	0.06	0.955
OS in Caucasians	4	2.07 (1.41, 2.73)	<0.05	0%	0.92	0.34	0.734	0.05	0.964
OS in Asians	1	3.79 (2.18, 6.35)	<0.05	–	–	–	–	–	–

OR, odds ratio; CI, confidence interval; OS, overall survival.

## Discussion

It is well established that oxygen is an electron acceptor that participates in most biological reactions and plays a central role in maintaining intracellular Adenosine Triphosphate (ATP) levels. Therefore, hypoxia in tissues and organs can prove to be a serious health condition. We also know that, although rapid proliferation and growth of tumor cells need large amounts of nutrients and oxygen, hypoxic regions are often found in tumor tissues (partial pressure of oxygen <10 mmHg) (45). Therefore, the response to hypoxia is vital to the growth of tumor cells. In fact, multiple adaptive pathways are activated to enable tumor cells to adapt to low oxygen and poor nutrition microenvironments (46). The physiological process of hypoxia in cancer cells involves the interplay of reactive oxygen species (ROS), hypoxia-induced signaling pathways, glucose metabolism, and prevailing oxygen tension (47). In many cancer patients, hypoxia commonly leads to a poor prognosis due to natural selection of oxygen-resistant cells and cells resistant to radiation and chemotherapy, which makes metastasis more likely (48). HIF-1α is a sensitive transcription factor that can sense oxygen in the cell’s microenvironment and upregulate the expression of collagen and extracellular matrix remodeling enzymes, promoting tissue fibrosis and leading to tumor cell migration (49). In in vitro studies, HIF-1α inhibitors significantly reduced HIF-1α activity and inhibited the migration and proliferation of liver tumor cells (50). The intracellular accumulation of HIF-1α protein increased anti-apoptotic activity in hypoxia and radiotherapy (51, 52). Moreover, HIF-1α combined with p65 and p300 forms a complex that can affect cell function (53). However, mechanistic studies on the role of HIF-1α in endometrial cancer cells or any related cell line have still not been conducted. Based on the above evidence, hypoxia and HIF-1α might be good therapeutic targets for oncotherapy.

Here, we analyzed relevant literature to investigate the immunohistochemical expression of HIF-1α in endometrial cancer. The frequency of overexpression of HIF-1α in endometrial cancer tissue was significantly higher than in normal tissues. In addition, the expression of HIF-1α was significantly higher in endometrial cancer patients with grade 2 and 3 tumors than those with grade 1 tumors, which was found in both Caucasians and Asians. Because significant heterogeneity was found in Asians, we used the random-effect model and conducted a sensitivity analysis. The results of the sensitivity analysis indicate that no individual study disproportionately affected the pooled overall results for Asian patients. In the included articles, we found two studies that report opposite results, suggesting that HIF-1α is a protective factor for tumor development, which might have led to significant heterogeneity among studies (28, 35). Further, overexpression of HIF-1α significantly promoted lymph node metastasis of tumor cells. However, the results of the subgroup analysis suggest that the association was only found in Asians. Because only two studies in Caucasians were included for the analysis of lymph node metastasis, the statistical power might be lower because of the smaller sample size. In addition, HIF-1α protein overexpression was significantly associated with myometrial invasion of endometrial tumor cells in Asians. In the subgroup analysis, heterogeneity clearly decreased, which might indicate the effect of ethnicity on heterogeneity among studies. Notably, we also found HIF-1α protein expression in many other human cancers, such as bladder, lung, and colorectal cancer (15–17). The Sivridis study on the correlation of HIF-1α with prognosis of endometrial cancer is the first study to investigate the role of HIF-1α protein expression in endometrial cancer patients (32). Subsequently, several clinical or mechanistic studies were performed to determine the effect of HIF-1α protein in endometrial cancer. However, HIF-1α levels in different endometrial cancer subgroups remain unknown, and future studies on the new molecular subtypes of endometrial cancer are needed. We also analyzed the association between HIF-1α protein expression and other clinical features, such as TNM (T: primary tumor; N: regional lymph nodes; M: distant metastasis) stage, FIGO (International Federation of Gynecology and Obstetrics) stage, PR (progesterone receptor) status, ER (estrogen receptor) status, and recurrence of endometrial cancer; no significant associations were observed. Three studies were included for tumor recurrence with one study reporting a positive result. Therefore, more studies need to be conducted to observe the role of HIF-1α in endometrial cancer recurrence.

The pooled HR shows that HIF-1α positive expression is significantly associated with poor prognosis in patients with endometrial cancer. Patients with high HIF-1α protein expression had a lower survival rate than those with low HIF-1α expression; high HIF-1 α levels indicate that endometrial cancer tissues are hypoxic. HIF-1α also predicates a poor prognosis in ovarian cancer (54). However, in lung and colorectal cancer, HIF-1α is not related with patient prognosis, indicating that HIF-1α plays distinct roles in different tumor types (55, 56). No significant heterogeneity and publication bias was found in the meta-analysis for prognosis. Thus, the overall results of the analysis could be considered reliable.

This analysis had several limitations. First, although 25 eligible studies were retrieved, only a few studies were included in each group after classification based on PR, ER, and recurrence. Second, some heterogeneity among studies was detected in the analysis for clinical features; heterogeneity in the subgroup analysis decreased but could not be eliminated. This might be attributed to differences in sampling or detection methods and future studies with larger sample sizes should be conducted. Third, the quality of antibodies used for IHC was not consistent, which might also have contributed to heterogeneity.

## Conclusion

In summary, although certain limitations exist, the results still demonstrate that HIF-1α overexpression is positively associated with high-grade tumors, lymphatic invasion, and myometrial invasion. Furthermore, overexpression of HIF-1α is a good predictor of poor prognosis of endometrial cancer patients. However, these results need to be further verified by well-designed studies with larger sample sizes. In addition, mechanistic studies on the role of HIF-1α in endometrial cancer should be conducted.

## Data Availability Statement

All datasets presented in this study are included in the article/**Supplementary Material**.

## Author Contributions

Conceived and designed the experiments: PZ, LS, XH. Performed the experiments: PZ, LS. Analyzed the data: PZ, LS. Contributed reagents/materials/analysis tools: PZ, LS, QR, QZ. Wrote the paper: PZ, LS, QZ. All authors contributed to the article and approved the submitted version.

## Conflict of Interest

The authors declare that the research was conducted in the absence of any commercial or financial relationships that could be construed as a potential conflict of interest.
